# Public Health, Hygiene and Bacteriology

**Published:** 1895-11

**Authors:** Ernest Wende

**Affiliations:** Health Commissioner of the City of Buffalo, N. Y.


### PUBLIC HEALTH, HYGIENE AND BACTERIOLOGY.

Conducted by ERNEST WENDE, M. D.,
Health Commissioner of the City of Buffalo, N. Y.

### September, 1895, Health Records.

By FRANKLIN C. GRAM, M. D., Registrar.

THE records show that there were 115 less deaths during this month than during the same month in 1894. This is an exceptionally great difference, and it is just as pertinent to ask what caused this great decrease as it would be to account for any sudden increase.

In the first place, the entire season has been a healthy one. Add to this the constant vigilance exercised by physicians, sanitarians, health authorities and the public at large, and we have a factor which must prove a very potent one and which is, at the same time, a powerful preventive against any epidemic.

A comparison with September, 1894, does not show a marked difference in any one class of diseases, but in all of them. They are herewith enumerated, the first figures being for 1894 and the second for 1895 respectively: Communicable diseases, 175, 136; development or decline, 13, 10; perverted or deficient nutrition, 62, 41; diseases of the nervous system, 53, 43 ; circulatory system, 31, 21 ; respiratory system, 24, 24 ; digestive system, 76, 53 ; urinary system, 16, 15 ; generative system of the female, 6, 7 ; osseous and muscular system, 1, 1 ; violence, 18, 19. Total deaths for September, 1894, 485 ; total deaths for September, 1895, 370.

This makes a difference of 115 deaths less than in September, 1894, and 166 less deaths than in 1893.

The contagious diseases reported for 1894 and 1895, respectively, were: measles, 2, 1 ; scarlet fever, 58, 24 ; diphtheria, 32, 54 ; consumption, 43, not reported last year.

There was no very great difference in the births and marriages, they being 643 and 646 of the former, and 227 and 233 of the latter for 1894 and 1895 respectively.

It is a remarkable fact that the mean temperature for the month was identical for both years—namely, 65° ; the mean barometer for 1894 was 29.34, and for 1895, 29.29 ; the total precipitation in 1894 was 5.59 inches, and 2.59 inches in 1895.

---

NURSING AS A PROFESSION.—Expend much time in earnest thought and weigh yourself and your capabilities before deciding to become a trained nurse. If you desire an easy profession select that of the washerwoman in preference. If you are a practical, business-like woman, and think of it as a profession which, when acquired, will yield you a good income in dollars and cents, and are willing to undergo two years of hospital service for the sake of acquiring it, and look at it from this standpoint only, you may possibly make a mechanical nurse. Your patients will not mourn your departure and you will never be called to visit the same household twice. If you are a romantic, novel-devouring maid, yearning to be known as a modern St. Elizabeth, and long to bathe weary brows and witness impossible death-bed scenes, and the like,—stay at home. You will remain at the hospital but a few days and you will be sadly disappointed. But if you are a strong, healthy woman, possessed of education, gentle breeding, a kind heart, determination, patience, and, above all, adaptability, and are willing to undertake whatever work is assigned you, and to face bravely whatever comes—if you are willing to forget your present station in life in order that you may become a useful woman and fit yourself for whatever fate the future holds in reserve for you, you will not regret the step you contemplate taking.– *From "The Training of a Nurse" in Demorest's Magazine for August.—Cleveland Med. Gazette.*

